# Venous blood gas in free-living eastern box turtles (*Terrapene carolina carolina*) and effects of physiologic, demographic and environmental factors

**DOI:** 10.1093/conphys/coy041

**Published:** 2018-07-25

**Authors:** Laura Adamovicz, Katie Leister, John Byrd, Christopher A Phillips, Matthew C Allender

**Affiliations:** 1 Wildlife Epidemiology Lab, Department of Veterinary Clinical Medicine 2001 S. Lincoln Ave., Urbana, IL 61802, USA; 2 Clinch River Environmental Studies Organization Oak Ridge, TN, USA; 3 Illinois Natural History Survey, Prairie Research Institute, 1816 S. Oak St., Champaign, IL 61820, USA

**Keywords:** Blood gas, chelonian, eastern box turtle, iSTAT, reptile, *Terrapene carolina carolina*

## Abstract

Sustainable wildlife populations depend on healthy individuals, and the approach to determine wellness of individuals is multifaceted. Blood gas analysis serves as a useful adjunctive diagnostic test for health assessment, but it is uncommonly applied to terrestrial reptiles. This study established reference intervals for venous blood gas panels in free-living eastern box turtles (*Terrapene carolina carolina*, *N* = 102) from Illinois and Tennessee, and modeled the effects of environmental and physiologic parameters on each blood gas analyte. Blood gas panels included pH, partial pressure of oxygen (pO_2_), partial pressure of carbon dioxide (pCO_2_), total carbon dioxide (TCO_2_), bicarbonate (HCO_3_^−^), base excess (BE) and lactate. Candidate sets of general linear models were constructed for each blood gas analyte and ranked using an information-theoretic approach (AIC). Season, packed cell volume (PCV) and activity level were the most important predictors for all blood gas analytes (*P* < 0.05). Elevations in PCV were associated with increases in pCO_2_ and lactate, and decreases in pH, pO_2_, HCO_3_^−^, TCO_2_ and BE. Turtles with quiet activity levels had lower pH and pO_2_ and higher pCO_2_ than bright individuals. pH, HCO_3_^−^, TCO_2_ and BE were lowest in the summer, while pCO_2_ and lactate were highest. Overall, blood pH was most acidic in quiet turtles with elevated PCVs during summer. Trends in the respiratory and metabolic components of the blood gas panel tended to be synergistic rather than antagonistic, demonstrating that either (1) mixed acid–base disturbances are common or (2) chelonian blood pH can reach extreme values prior to activation of compensatory mechanisms. This study shows that box turtle blood gas analytes depend on several physiologic and environmental parameters and the results serve as a baseline for future evaluation.

## Teaser

Blood gas analysis serves as a useful adjunctive test for health assessment in wildlife, but is infrequently utilized in free-living reptiles. This study demonstrates that box turtle blood gas analytes depend on several physiologic and environmental parameters including season, temperature, packed cell volume and activity level.

## Introduction

Blood gas analysis facilitates the clinical assessment of acid–base status, oxygenation and ventilation. It serves as a useful adjunctive diagnostic test for the assessment of individual animal health (Keller *et al.*, 2012; Stacy *et al.*, 2013; 2017). In wildlife, blood gas panels are employed primarily to evaluate the physiologic effects of capture/restraint techniques (e.g. Innis *et al.*, 2014; Harms *et al.*, 2016) and sedative/anesthetic protocols (e.g. Buss *et al.*, 2015; Spriggs *et al.*, 2017); with relatively infrequent application for population health assessment (e.g. Lewbart *et al.*, 2014; 2015; Páez-Rosas *et al.*, 2016; Muñoz-Pérez *et al.*, 2017; Ratliff *et al.*, 2017). However, increasing availability and affordability of point-of-care analyzers has boosted reporting of blood gas analytes from free-living wildlife populations in recent years (Stoot., 2014). The establishment of baseline physiologic parameters aids in complete characterization of population wellness, and may enhance conservation efforts (Cooke *et al.*, 2013; Madliger *et al.*, 2016).

Species which experience frequent respiratory and acid–base challenges are especially likely to benefit from blood gas analysis (Camacho *et al.*, 2013). For example, critically-ill sea turtles commonly experience significant acid–base abnormalities and metabolic compromise (Innis *et al.*, 2007, 2009; Anderson *et al.*, 2011; Keller *et al.*, 2012; Camacho *et al.*, 2013, 2015). Blood gas panels are used to guide veterinary management of these cases (Innis *et al.*, 2007, 2009; Camacho *et al.*, 2015), and form a component of mortality prediction indices for cold-stunned Kemp’s ridley sea turtles (*Lepidochelys kempii*) (Keller *et al.*, 2012; Stacy *et al.*, 2013, 2017). They have also been utilized to investigate the sublethal effects of bycatch (Harms *et al.*, 2003; Innis *et al.*, 2010), and the coupling of blood gas abnormalities with direct mortality data has contributed to trawling regulation protecting free-living sea turtles (Henwood and Stuntz, 1987; Stabenau and Heming, 1991). Similarly, blood gas panels paired with behavioral analyses have been used to justify the use of turtle exclusion devices and/or altered fyke netting protocols for freshwater fisheries (Larocque *et al.*, 2012; Stoot *et al.*, 2013; LeDain *et al.*, 2013). While blood gas panels have contributed to direct conservation action for aquatic chelonians, they are infrequently applied to free-living terrestrial species.

Eastern box turtles (*Terrapene carolina carolina*) are in decline due to a combination of factors including habitat loss, road mortality, subsidized predation and disease (Dodd., 2001). Several disease presentations in box turtles have respiratory manifestations including pneumonia in a hatchling turtle infected with Terrapene herpesvirus 1 (Sim *et al.*, 2015), rhinitis with serous to mucopurulent nasal discharge in turtles infected with *Mycoplasma* sp. (Feldman *et al.*, 2006), and a constellation of signs from nasal discharge to respiratory distress associated with ranavirus infection (Johnson *et al.*, 2008). Non-specific upper respiratory disease is also common in box turtles presented to wildlife rehabilitators (Sack *et al.*, 2017). With the exception of ranavirus, which causes high morbidity and mortality in box turtles, the effects of the other diseases on population health are currently uncharacterized (De Voe *et al.*, 2004; Johnson *et al.*, 2008; Sim *et al.*, 2016). Evaluating infection status in conjunction with complete health assessments, including blood gas analysis, may elucidate the effects of these respiratory pathogens at the population level. However, the dramatic physiologic changes associated with growth, reproduction and brumation in reptiles translate to extreme variability in bloodwork parameters based on season, sex, age class and reproductive status (e.g. Anderson *et al.*, 1997; Flower *et al.*, 2014; Rose and Allender, 2011; Tamukai *et al.*, 2011). It is therefore important to define the range of expected normal values in free-living populations prior to applying blood gas panels for clinical health assessment.

The objectives of this study were to (1) assess the impacts of physiologic, demographic and environmental factors on venous blood gas parameters in eastern box turtles and (2) to generate venous blood gas reference intervals appropriately partitioned over a range of physiologically relevant environmental conditions. The specific biological hypotheses were that blood gas parameters would be non-directionally influenced by environmental factors including season and temperature, and by physiologic factors including activity level and packed cell volume (PCV).

## Methods

### Animal populations

Eastern box turtles were captured using human and canine search teams in Oak Ridge, Tennessee (36.008°N, −84.22392°W), and Vermilion County, Illinois including the Middle Fork State Fish and Wildlife Area (40.2595°N, −87.7939°W), Kickapoo State Park (40.01167°N, −87.7359°W), Forest Glen Nature Preserve (40.0118°N, −87.5653°W) and Kennekuk Cove County Park (40.2085°N, −87.7232°W) during the 2014 active season. Turtles were sampled during the months of May (‘Spring’), June/July (‘Summer’), and September (‘Fall’). Capture locations were recorded using global positioning software (GPS) via eTrek Vista HCx hand held devices (Garmin International Inc., Olathe, KS, USA). Turtles were placed in individual cloth bags and transported in backpacks to a field sampling station for physical examination and blood collection. Following sampling, each turtle was released at its exact site of capture. Procedures were approved by the University of Illinois Institutional Animal Use and Care Committee (Protocols #13 061 and #15 017).

### Sample collection and processing

All sample collection occurred within 2 h of capture. Each animal was assigned a permanent ID via shell notching, and the date, weight, size (straight carapace length, width, and height; anterior and posterior plastron length), approximate age (annuli estimate), sex and age class were recorded. Sex was determined based on iris color, plastron concavity and positioning of the cloacal opening (Dodd., 2001). Turtles with a carapace length less than 9 cm (3.5 in) and an annuli count less than or equal to seven were characterized as juveniles, all others were classified as adults. Physical examinations were performed, noting visual appearance of the eyes, nose, tympanic membranes, oral cavity, appendages, shell, cloaca and integument. Each of these systems was coded as ‘normal’ or ‘abnormal.’ Activity level was assessed as ‘bright’ (moving or active) or ‘quiet’ (boxed up or minimal movement).

Whole blood (<0.8% body weight) was collected from the subcarapacial sinus using a 22-gauge, 1.5-inch needle and 3-ml syringe (adults) or a 25-gauge, ¾ inch needle and 1-ml syringe (juveniles). Following collection, blood was immediately loaded into a CG4+ blood gas cartridge (iSTAT, Abbott, North Chicago, IL) and analyzed (iSTAT 2, Abbott) for the following parameters: pH, partial pressure of oxygen (pO_2_), partial pressure of carbon dioxide (pCO_2_), total carbon dioxide (TCO_2_), bicarbonate (HCO_3_^−^), base excess (BE), and lactate. The iSTAT system heats blood samples to 37°C and measures pH and pCO_2_ using direct potentiometry, while pO_2_ is measured amperometrically. HCO_3_^−^, TCO_2_ and BE are calculated using human-derived algorithms (CLSI, 2001). Heparinized, clotted and lymph-diluted samples were excluded from analysis.

Values for pH, pO_2_ and pCO_2_ were corrected based on average air temperature (*T*_A_), and HCO_3_^−^ and TCO_2_ were re-calculated using the following formulae (Stabenau and Heming, 1993; CLSI 2001; Anderson *et al.* 2011):

$$\begin{array}{c} pH\left(T_{A}\right)\;=\;{\mathrm{pH}}_{I}–0.0147\times \left(T_{A}–37\right)+0.0065\\ \times \left(7{.4–\mathrm{pH}}_{I}\right){\times (T}_{A}–37) \end{array}$$

$$pO_{2}\left(T_{A}\right)\;=\;{\mathrm{pO}}_{2I}{\times 10}^{-0.0058*\left(\mathrm{TA}–37\right)}$$

$$pCO_{2}\left(T_{A}\right)\;=\;{\mathrm{pCO}}_{2I}\times 10^{0.0019*\left(\mathrm{TA}–37\right)}$$

$$HC{O_{3}}^{-}\left(T_{A}\right)\;=\;{\alpha \mathrm{CO}}_{2}\times {\mathrm{pCO}}_{2}\left(T_{A}\right)\times 10^{\left(\mathrm{pH}\left(\mathrm{TA}\right)–\mathrm{pKa}\right)}$$

$$TCO_{2}\left(T_{A}\right)\;=\;{{\mathrm{HCO}}_{3}}^{-}\left(T_{A}\right)+\left({\alpha \mathrm{CO}}_{2}\times {\mathrm{pCO}}_{2}\left(T_{A}\right)\right)$$

$$\begin{array}{c} \alpha CO_{2}\;=\;9.174\times 10^{-2}–3.269\times 10^{-3}\times T_{A}\\ +6.364\times 10^{-5}\times {T_{A}}^{2}–5.378\times 10^{-7}\times {T_{A}}^{3} \end{array}$$

$$\begin{array}{c} pKa\;=\;6.398–1.341\times 10^{-2}\times T_{A}+2.282\times 10^{-4}\\ \times T_{A}^{2}–1.516\times 10^{-6}\times T_{A}^{3}\;–\;\log(1.011\\ +10^{\mathrm{pH}\left(\mathrm{TA}\right)+0.011\times \mathrm{TA}–10.241}+10^{\mathrm{pH}\left(\mathrm{TA}\right)+0.001\times \mathrm{TA}–8.889}) \end{array}$$

PCV was determined using heparinized microhematocrit tubes (Jorgensen Laboratories, Inc., Loveland, CO 80 538) centrifuged at 14 500 rpm for 5 min. Environmental temperature data (daily minimum, maximum, and mean air temperature) were obtained from NOAA weather stations nearest to the study site for the dates corresponding to each search.

### Statistical methods

All statistical assessments were performed in R at an alpha value of 0.05 (R Core Team, 2013). Descriptive statistics (mean, median, range, standard deviation, and 10% and 90% percentiles) were tabulated for all continuous variables. Distribution was assessed visually using box plots and statistically using the Shapiro–Wilk test. Data transformation was pursued if needed to support statistical assumptions during modeling. Differences in categorical variables (sex, age class) between study sites and states were evaluated using Fisher’s exact tests. Sex ratios were evaluated using binomial tests (expected ratio 0.5). Differences in continuous variables (weight, PCV) between study sites, states and seasons were assessed using general linear models. For each model, the assumption of normally distributed error in the dependent variable was evaluated using boxplots, histograms and Q–Q plots of residual values. The assumption of homoscedasticity was assessed using plots of actual versus fitted residuals and Levene’s tests. The impact of influential values was assessed using Cook’s Distance plots. Post hoc between group differences were evaluated using the contrasts function in the lsmeans package (Lenth, 2016).

A directed acyclic graph (DAG) was generated to demonstrate the expected relationships among measured predictors and their effect on blood gas parameters. This diagram was used to identify potential confounding variables (variables which influence both the predictor of interest and the response variable) and structure statistical analyses (Joffe *et al.*, 2012). The effects of confounding variables on parameter estimates were controlled using multivariable linear regression. Continuous predictor variables were assessed for multicollinearity using Pearson’s correlation coefficient, with *r* > 0.5 considered ‘strongly’ correlated. Strongly correlated predictor variables were not included together in future models. Following this, sets of univariate and multivariate candidate models were constructed for each blood gas parameter and ranked using information-theoretic approaches with the AICcmodavg package (Mazerolle, 2017).

Reference intervals for each blood gas parameter were partitioned based on the results of general linear modeling and constructed according to American Society for Veterinary Clinical Pathology guidelines (Friedrichs *et al.*, 2012). Turtles with evidence of active disease processes (ocular/nasal discharge, oral plaques, open-mouth breathing, etc.) or non-healed traumatic injuries were excluded from the reference interval dataset. Outliers were visually identified using box plots and excluded using Horn’s method (Horn *et al.*, 2001). The mean, standard deviation, median and range were determined for each parameter. Data were evaluated for normality using the Shapiro−Wilk and Kolmorgorov–Smirnov tests. The nonparametric method was used to generate 95% reference intervals for each blood gas parameter based on the observations of Le Boedec *et al.* (2016). Ninety percent confidence intervals were generated around the upper and lower bounds of each reference interval using nonparametric bootstrapping with 5000 replicates. The width of the confidence intervals (WCI) was compared to the total width of the reference interval (WRI) to infer the need for a larger sample size (improved n is recommended when WCI/WRI > 0.2). All reference interval generation was performed using the referenceIntervals package.

## Results

### Sample population

A total of 102 eastern box turtles were included in this study. The sampled populations are described by season, state, sex, age class and habitat in Table 1. There were no significant differences in sex or age class distribution between sites or states.

**Table 1:** Venous blood gas sample size by state, sex, age class and season in eastern box turtles (*Terrapene carolina carolina*).

	Spring	Summer	Fall
**State**			
Illinois	20	19	16
Tennessee	20	23	4
**Sex**			
Male	23	19	12
Female	15	21	5
Unknown	2	1	3
**Age class**			
Adult	37	40	17
Juvenile	3	2	3

### Physical exam

Most turtles had a quiet activity level *n* = 66; 67.3%), but some were bright (*n* = 32; 32.7%). Carapacial lesions were the most common physical exam abnormality (*n* = 9; 9%), followed by asymmetrical nares (*n* = 3; 3%), plastron lesions (*n* = 2; 2%), a nodule on the hard palate (*n* = 1; 1%), unilateral pthisis bulbi (*n* = 1; 1%), necrotic bridge fracture (*n* = 1; 1%), open-mouth breathing (*n* = 1; 1%), and a combination of ocular swelling, nasal discharge and diarrhea (*n* = 1; 1%). The last three aforementioned turtles with active lesions were excluded from the dataset used for reference interval generation.

### Physiologic and environmental parameters

Continuous physiologic parameters are summarized in Table 2. Illinois turtles had a greater mass than Tennessee turtles (effect size = 137 g, *P* < 0.0001). Within Illinois, EBT at Collison were significantly heavier than those at Forest Glen Nature Preserve (effect size = 83 g, *P* = 0.04). PCV in EBT from TN was significantly higher than in IL turtles (effect size 4.1%, *P* = 0.004).

**Table 2:** Descriptive statistics for continuous physiologic variables from eastern box turtles (*Terrapene carolina carolina*) sampled for venous blood gas analysis.

	Weight (g)	*T*_A_ (^°^C)	PCV (%)
**Mean**	429	20.97	25
**Median**	411	20.83	25
**SD**	143	3.82	8
**Range**	41–761	13.89–26.67	6–45.5

*T*_A_ = average air temperature, PCV = packed cell volume.

Relationships between environmental and physiologic variables were explored statistically. Average daily air temperature (*T*_A_, °C) was significantly associated with season (*P* < 0.0001). Spring temperatures were lower than summer temperatures (effect size = 5.47°C, *P* < 0.0001), summer temperatures were higher than fall temperatures (effect size = 7.82°C, *P* < 0.0001), and fall temperatures were lower than spring temperatures (effect size = 2.34°C, *P* = 0.0001). PCV was positively associated with *T*_A_ (*r* = 0.3, *P* = 0.0034) and dependent upon season (*P* < 0.0001), with higher PCV values in the summer compared to both spring (effect size = 7%, *P* < 0.0001) and fall (effect size = 4.7%, *P* = 0.012). PCV tended to be higher in quiet turtles compared to bright individuals, though this relationship was not statistically significant (effect size = 2.9%, *P* = 0.059).

### Blood gas data assessment

iSTAT errors resulted in missing values for BE (*n* = 2), and lactate (*n* = 1). During initial assessment of blood gas parameter distribution, three turtles were identified with outlier values for two or more blood gas parameters. These animals were fully assessed and determined to have significant pathologic acid–base disturbances, as such they were excluded from modeling and reference interval generation. Blood gas values for these excluded animals are provided in Supplementary Table S1.

### Blood gas modeling

**Figure 1:**
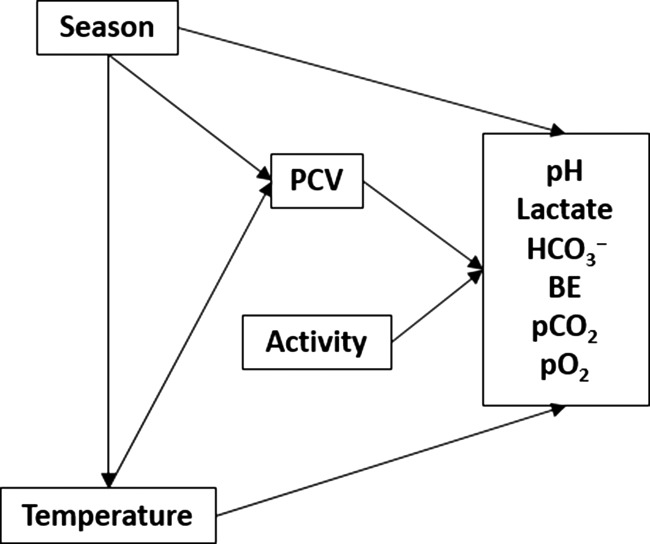
Directed acyclic graph depicting the relationships between venous blood gas parameters and their predictors in eastern box turtles (*Terrapene carolina carolina*).

**Figure 2:**
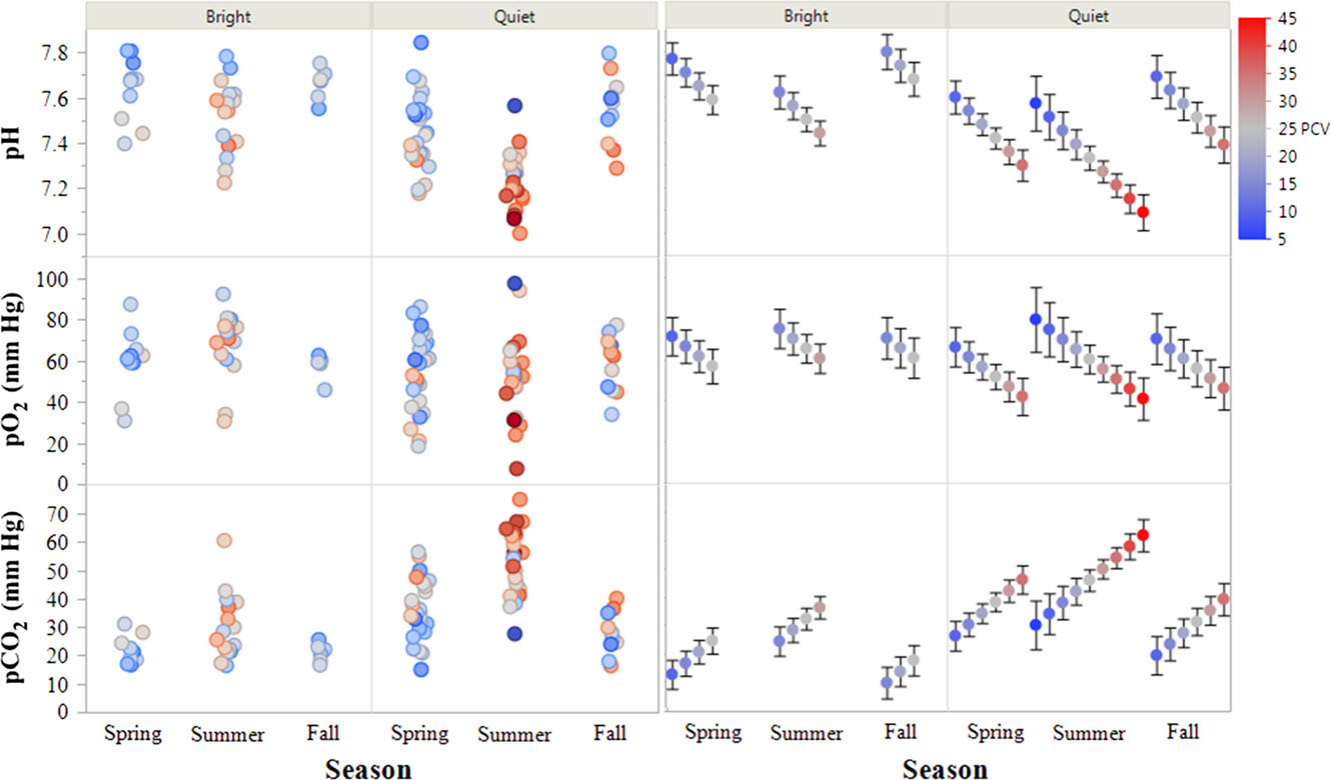
Measured and predicted venous blood gas values in free-living eastern box turtles (*Terrapene carolina carolina*). Left panels: measured values. Right panels: values predicted from general linear models containing the additive effects of activity level, season, and packed cell volume (PCV, %), ± 95% confidence intervals of the estimates.

**Figure 3:**
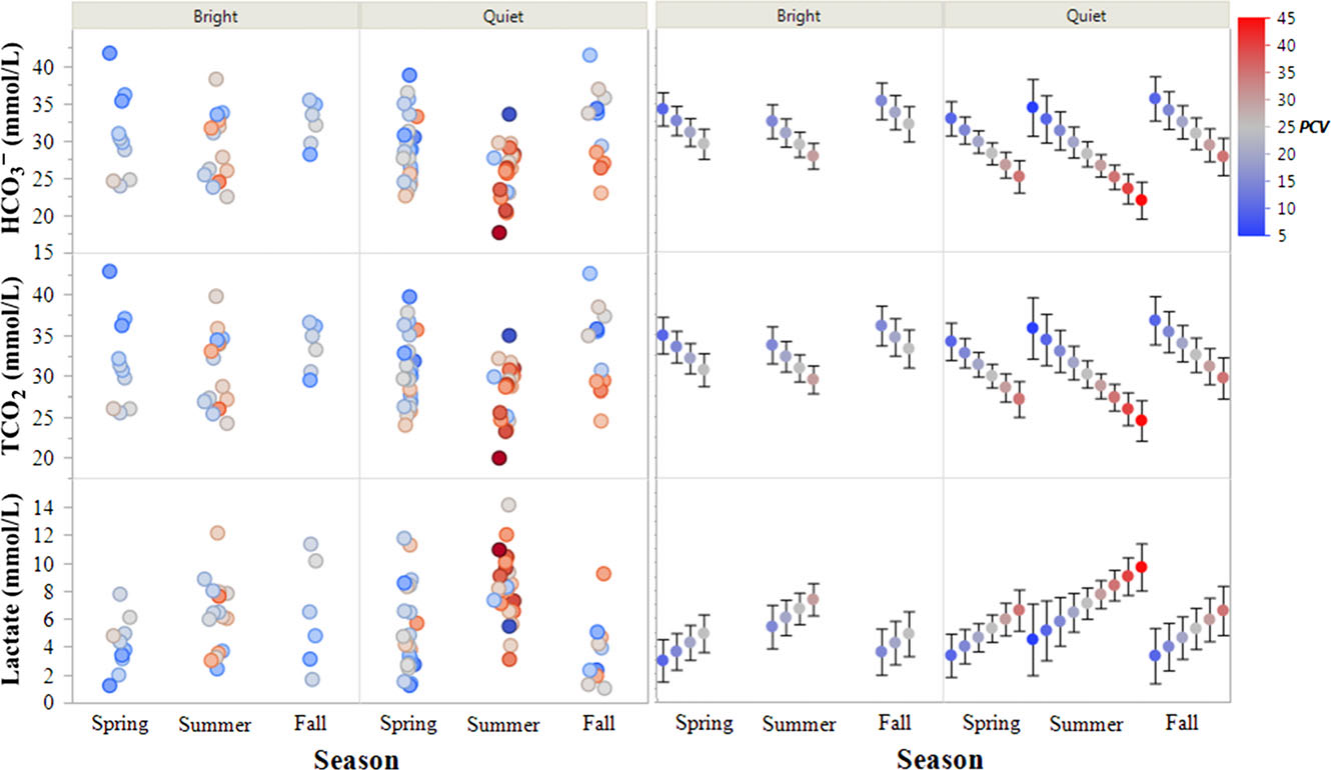
Measured and predicted venous blood gas values in free-living eastern box turtles (*Terrapene carolina carolina*). Left panels: measured values. Right panels: values predicted from general linear models containing the additive effects of activity level, season, and packed cell volume (PCV, %), ± 95% confidence intervals of the estimates.

Modeling of blood gas analytes was pursued to fulfill two main goals: (1) Determine the most precise effect estimates for each significant predictor variable and (2) Identify the most parsimonious models to predict blood gas values. To address Goal 1, models of each blood gas analyte were structured and assessed inclusive of confounding variables and without intervening variables (variables on the causal pathway between the predictor of interest and the response variable) based on a DAG (Fig. 1). General relationships are displayed in Figures 2 and 3; final effect estimates are shown in Supplementary Table S2.

pH was negatively associated with *T*_A_ (*P* < 0.001) and PCV (*P* < 0.0001). Lower pH was also observed in quiet turtles (effect size = 0.19, *P* < 0.0001) and the lowest pH values were observed in the summer compared to both spring (effect size = 0.13, *P* = 0.003) and fall (effect size = 0.22, *P* = 0.001). pO_2_ was negatively associated with PCV (*P* = 0.001) and lower in quiet turtles (effect size = 9.3 mm Hg, *P* = 0.018). pCO_2_ was positively associated with *T*_A_ (*P* < 0.0001) and PCV (*P* < 0.0001). It was higher in quiet turtles (effect size = 15 mm Hg, *P* < 0.001) and the highest values were observed in the summer compared to both spring (effect size = 10, *P* = 0.002) and fall (effect size = 17.5, *P* < 0.001)). HCO_3_^−^ was negatively associated with *T*_A_ (*P* < 0.0001) and PCV (*P* < 0.0001). It was lower in summer than fall (effect size = 4.37mmol/l, *P* = 0.009). TCO_2_ was negatively associated with *T*_A_ (*P* = 0.0005) and PCV (*P* < 0.0001) and was dependent upon season, with lower values in the summer compared to the fall (effect size = 3.89 mmol/l, *P* = 0.02). Lactate was positively associated with *T*_A_ (*P* = 0.017) and PCV (*P* = 0.009), and was highest in summer compared to spring (effect size = 2.36 mmol/l, *P* = 0.0003) and fall (effect size = 2.72 mmol/l, *P* = 0.0008). BE was negatively associated with T_A_ (*P* = 0.002) and PCV (*P* < 0.0001). It was lowest in the summer compared to spring (effect size = 3.07 mmol/l, *P* = 0.006) and fall (effect size = 4.71 mmol/l, *P* = 0.0009).

To address Goal 2, sets of univariate and multivariate candidate models constructed from a common dataset were ranked using information-theoretic model selection procedures. Case-wise deletion was used to address missing data. One record was removed due to a missing PCV, three records were removed due to missing activity levels, and one record was removed due to missing both PCV and activity level entries. Records with missing BE and lactate values due to iSTAT errors were also removed. Season was included in place of *T*_A_ in all models due to the highly correlated nature of these two variables and the antecedent position of Season relative to *T*_A_ in the DAG. All biologically important predictor variables were included for model selection regardless of statistical significance. The results of predictive model construction and selection are displayed in Table 3 and Figures 2 and 3.

**Table 3:** Model selection criteria for venous blood gas parameters in eastern box turtles (*Terrapene carolina carolina*)

Parameter	Model	N	K	AIC_c_	ΔAIC_c_	w_i_
pH	Activity + PCV + Season	96	6	−104.56	0	1
	PCV + Season	96	5	−77.98	26.57	0
	Activity	96	3	−49.63	54.93	0
	Season	96	4	−45.56	59.00	0
	Null	96	2	−29.32	75.24	0
pO_2_ (mm Hg)	PCV + Season	96	5	761.99	0	0.53
	Activity + PCV + Season	96	6	762.45	0.46	0.42
	Activity	96	3	767.21	5.22	0.04
	Null	96	2	770.52	8.53	0.01
	Season	96	4	774.37	12.38	0
pCO_2_ (mm Hg)	Activity + PCV + Season	96	6	655.72	0	1
	PCV + Season	96	5	688.59	32.87	0
	Activity	96	3	713.08	57.35	0
	Season	96	4	717.21	61.49	0
	Null	96	2	736.06	80.34	0
HCO_3_^−^ (mmol/l)	PCV + Season	96	5	509.08	0	0.55
	Activity + PCV + Season	96	6	509.48	0.40	0.45
	Season	96	4	530.62	21.54	0
	Activity	96	3	533.91	24.82	0
	Null	96	2	536.32	27.24	0
TCO_2_ (mmol/l)	PCV + Season	96	5	506.33	0	0.69
	Activity + PCV + Season	96	6	507.95	1.62	0.31
	Season	96	4	523.50	17.17	0
	Activity	96	3	526.83	20.50	0
	Null	96	2	527.43	21.10	0
Lactate (mmol/l)	PCV + Season	96	5	430.56	0	0.71
	Activity + PCV + Season	96	6	432.49	1.93	0.27
	Season	96	4	437.64	7.09	0.02
	Null	96	2	450.64	20.09	0
	Activity	96	3	452.12	21.56	0
BE (mmol/l)	PCV + Season	88	5	509.24	0	0.57
	Activity + PCV + Season	88	6	509.77	0.53	0.43
	Season	88	4	534.25	25	0
	Activity	88	3	544.18	34.94	0
	Null	88	2	545.01	35.76	0

AIC_c_ = AIC corrected for sample size, ΔAIC_c_ = difference compared to the smallest AIC_c_ value, w_i_ = Akaike weight.

For pH and pCO_2_, the models containing the additive effects of all predictors identified in the original DAG garnered the most support with Akaike weights of 1. For lactate and TCO_2_, PCV + Season was the most parsimonious model with Akaike weights of 0.69–0.71. There was high model selection uncertainty for the remaining blood gas parameters, but they all shared the same top two models which accounted for ≥0.95 of the Akaike weights: PCV + Season and the full additive model (PCV + Season + Activity Level). Objectively, top models had adjusted *R*^2^ values from 0.12 to 0.62 and *P*-values from 0.002 to <0.0001. Subjectively, models explained a fair-moderate degree of variability in the data, with decreased explanatory power at extreme values (Figs 2 and 3).

### Venous blood gas reference intervals

Reference intervals for blood gas parameters were partitioned based on season. Statistical differences were not identified based on sex or age class, so data from all apparently healthy animals was combined for reference interval generation. Excluded data included values missing due to the iSTAT errors mentioned above and outliers: HCO_3_^−^ (17.7 mm Hg in summer) and lactate (14.17 mmol/l in summer). Summary data, reference intervals and 90% CI of the reference interval bounds are reported in Table 4. The WCI/WRI ratios indicate that the reference interval bounds for HCO_3_^−^ in the spring and TCO_2_ in the spring may gain precision with a larger sample size. Reference intervals were not calculated for the fall sampling period due to sample size limitations, per ASVCP recommendations. Histograms showing the distribution of all data used to calculate reference intervals are available as Supplementary Figures S1–S3.

**Table 4:** iSTAT venous blood gas reference intervals for eastern box turtles (*Terrapene carolina carolina*) in the spring, summer, and fall

Parameter	Season	N	Mean	SD	Median	Min	Max	Distribution	Reference Range	90% CI LB	90% CI UB
pH	Spring	38	7.49	0.18	7.49	7.18	7.84	G	7.18–7.84	7.16–7.18	7.84–7.88
	Summer	41	7.36	0.2	7.33	7.00	7.78	G	7.00–7.78	6.92–7.01	7.78–7.88
	Fall	17	7.59	0.14	7.60	7.29	7.80	G	NA	NA	NA
pO_2_ (mm Hg)	Spring	38	57	19	60	18	87	G	18–87	10–18	87–90
	Summer	41	58	21	59	8	98	G	8–97	0–9	97–103
	Fall	17	58	11	59	34	77	G	NA	NA	NA
pCO_2_ (mm Hg)	Spring	38	32.8	12.1	31.2	15.1	64.9	G	15.1–64.9	13.0–15.1	64.9–74.6
	Summer	41	43.9	16.8	42.8	16.5	75.1	G	16.5–74.7	12.4–16.6	74.4–82.4
	Fall	17	25.3	7.0	24.7	16.4	40.2	G	NA	NA	NA
HCO_3_ (mmol/l)	Spring	38	29.4	4.8	28.8	22.6	41.8	G	22.6–41.8	22.3–22.6	41.8–47.1^a^
	Summer	40	28.2	5.1	27.7	17.7	40.6	G	20.4–40.6	18.4–20.4	40.5–42.9
	Fall	17	32.0	4.6	33.5	23.0	41.5	G	NA	NA	NA
TCO_2_ (mmol/l)	Spring	38	31	4.7	30	24	43	G	24–43	23–24	43–48^a^
	Summer	41	30	5.0	29	20	43	G	20–43	17–20	43–46
	Fall	17	33	4.5	35	25	43	G	NA	NA	NA
Lactate (mmol/l)	Spring	38	4.97	2.84	4.25	1.23	11.78	NG	1.23–11.78	1.08–1.23	11.78–13.06
	Summer	40	7.40	2.72	7.63	2.43	14.17	G	2.45–12.16	1.77–2.46	12.15–13.38
	Fall	16	4.61	3.21	4.08	1.04	11.36	NG	NA	NA	NA
BE (mmol/l)	Spring	37	1.9	4.9	2	−7	11	G	−7–11	−10, –7	11–13
	Summer	40	−0.5	5.6	−1	−13	14	G	−13–14	−17, –13	14–19
	Fall	17	3.5	4.2	4	−5	10	G	NA	NA	NA

^a^ WCI/WRI > 0.2.

SD = standard deviation, NG = non-Gaussian distribution, G = Gaussian distribution, CI = confidence interval, LB = lower bound of the reference interval, UB = upper bound of the reference interval.

## Discussion

This study determined seasonal reference intervals for venous blood gas parameters in eastern box turtles across the active season, assessed environmental and physiologic predictors of blood gas status, and built predictive models for blood gas analytes. The results represent an important first step towards understanding blood gas analysis in free-living chelonians, and generate several important research questions for further study.

### Expected associations between predictors and blood gas parameters: the directed acyclic diagram

The directed acyclic diagram which guided modeling in this study was based on previous research into blood gas parameters in reptiles. Factors such as temperature and exercise level have direct effects on chelonian blood gas parameters, while season and PCV can indirectly influence blood gases (Gatten., 1974; Toledo *et al.*, 2008; da Silva *et al.*, 2013; Malte *et al.*, 2014).

In closed systems, increasing temperature decreases the solubility of CO_2_ and O_2_ in plasma (αCO_2_, αO_2_, respectively), decreases (typically) hemoglobin’s affinity for O_2_, and decreases the pK of buffering systems (pK_Pr_, pK_HCO_3__^−^), leading to a decrease in pH and an increase in pO_2_ and pCO_2_. These changes are tightly coupled by the Bohr/Haldane effect (Malte *et al.*, 2014). In ectothermic vertebrates, temperature directly affects pH, pO_2_, and pCO_2_ due to its effect on metabolic rate (da Silva *et al.*, 2013; Malte *et al.*, 2014). Increasing temperature increases oxygen consumption, oxygen uptake (VO_2_) and pulmonary ventilation (*V*_E_), but decreases the air convection requirement (*V*_E_/VO_2_) resulting in relative hypoventilation. This elevates the concentration of alveolar CO_2_, which in turn increases arterial CO_2_ and decreases pH (da Silva *et al.*, 2013). Temperature was placed antecedent to all blood gas parameters in the DAG to represent these relationships.

Season can affect reptile metabolic rates independently of temperature, potentially due to differences in light cycle, resource availability, and/or reproductive status (Toledo *et al.*, 2008). This may lead to seasonal changes in blood gas parameters (Christopher *et al.*, 1999). In the DAG, season was placed antecedent to temperature and all blood gas parameters.

Exercise directly affects oxygen consumption and lactate production in chelonians, and can be expected to influence blood gas parameters (Gatten, 1974; Bagatto and Henry, 1999). We were unable to directly assess exercise level in the turtles, so activity level (whether or not the turtle was moving upon examination) was used as a proxy variable in the DAG and was placed antecedent to all blood gas parameters.

PCV, an indicator of red blood cell concentration, is highly correlated to the concentration of hemoglobin, which serves as the oxygen carrying metalloprotein in vertebrates. PCV has been demonstrated to affect pO_2_ due to its role in oxygen carrying capacity, though its degree of importance depends on temperature, oxygen solubility, and the level of affinity of hemoglobin for O_2_ (Malte *et al.*, 2014). PCV was placed antecedent to all blood gas parameters in the DAG.

Relationships between predictor variables must also be considered for modeling purposes. Temperature is clearly influenced by season. This was demonstrated in the DAG by placing temperature on a causal pathway between season and all blood gas parameters. PCV is positively associated with temperature in loggerhead sea turtles (*Caretta caretta*), potentially related to temperature-dependent rates of erythropoiesis (Kelly *et al.*, 2015). Season also affects PCV in chelonians, with higher values typically observed during summer months (Christopher *et al.*, 1999; Chung *et al.*, 2009; Yang *et al.*, 2014). These relationships were illustrated in the DAG by creating causal pathways from season and temperature to PCV.

### Predictor variables not included in the DAG

Additional important causal factors in blood gas analysis are related to cardiorespiratory function. Turtles have a three-chambered heart with an incompletely-separated ventricle which permits admixture of oxygenated and unoxygenated blood. The net direction of blood flow within the heart can be right-to-left (away from the pulmonary circulation) or left-to-right (towards the pulmonary circulation). The direction of the shunt is vagally mediated, with the L–R direction predominating during periods of ventilation (with a corresponding increase in heart rate) and the R–L direction dominating during periods of apnea (with a corresponding decrease in heart rate), likely representing an evolutionary mechanism to cope with diving (Shelton and Burggren, 1976; White *et al.*, 1989; Hopkins *et al.*, 1996; Wang and Hicks, 1996). Intracardiac shunting has been demonstrated to affect blood gas parameters, with R–L shunts associated with lower arterial pO_2_ (Hicks and Wang, 1999; Platzack and Hicks, 2001). However, there is no non-invasive way to evaluate intracardiac shunting in chelonians. The closest measurable proxy variable is heart rate, which was not evaluated in this study, but should be considered in future research efforts.

Turtle respiratory patterns are characterized by alternating periods of ventilation and apnea (Burggren and Shelton, 1979). In box turtles, lung ventilation is primarily due to the function of the oblique abdominis and transverse abdominis muscles, with potential contribution from limb-pumping motions at rest (Landberg *et al.*, 2003). Respiratory rate was not recorded during field work due to the difficulty associated with accurate measurement of this variable in boxed turtles. Digestion also elevates metabolic rate and can influence blood gas parameters in reptiles (Arvedsen *et al.*, 2005). However, as our study subjects were wild, their ingestion history was unknown. As neither cardiorespiratory rates nor digestive status were measured in this study, they were not included in the DAG, despite their potential as biologically important predictor variables.

### Box turtle blood gas modeling

Most of the relationships depicted in the final DAG were statistically supported. As expected, PCV was positively associated with temperature and dependent upon season, with the highest values in summer. This finding is in agreement with previous research on the hematology of Asian yellow pond turtles (*Ocadia sinensis*), yellow-marginated box turtles (*Cuora flavomarginata*), loggerhead sea turtles, and desert tortoises (*Gopherus agassizii*) (Christopher *et al.*, 1999; Chung *et al.*, 2009; Yang *et al.*, 2014; Kelly *et al.*, 2015). The relationship between PCV and activity level was borderline significant, with quieter turtles having higher PCV values. This may be consistent with a stress response. When faced with a threatening stimulus, most animals have the option of ‘fight’ or ‘flight,’ but box turtles have a unique third option: retract the head and limbs within the shell and close the hinge to hide. This is the most frequent response to a perceived threat in box turtles (Smith and de Carvalho, 1985). A transient elevation in PCV is another component of the reptilian stress response (Sturbaum and Bergman, 1981; Sturbaum, 1981; Franklin *et al.*, 2003). Taken together, an elevated PCV in boxed or partially-boxed turtles may be indicative of stress.

pH decreased and pCO_2_ increased with increasing air temperatures, and these changes were most extreme during the summer months. This follows the trend of other ectothermic vertebrates, and is likely driven by an increased metabolic rate (da Silva *et al.*, 2013). HCO_3_^−^, TCO_2_ and BE, all indicators of the metabolic component of acid–base status, had a negative association with temperature and were lowest in summer. The opposite relationship was identified for lactate. While anion gap was not determined in this study, the combination of lower pH, HCO_3_^−^, TCO_2_ and BE with elevated lactate suggests a relative titrational metabolic acidemia in eastern box turtles during the summer compared to spring and fall. Elevated lactic acid production in the summer is likely secondary to increased activity levels associated with foraging, mating and nesting behaviors (Gatten, 1974; Bagatto and Henry, 1999). Concurrent elevation in pCO_2_ may indicate a mixed acidemia driven by the decreased air convection requirement associated with elevated temperature (da Silva *et al.*, 2013).

This is an interesting finding because typically changes in the respiratory and metabolic components of pH balance oppose each other in order to maintain normal physiologic functions. However, in apparently healthy free-living box turtles, it appears that these components are working in an additive fashion to drive pH down in summer months. Furthermore, this acid–base change is not large enough to activate compensatory mechanisms, indicating that box turtle acid–base balance may be naturally regulated within wide limits. This information has direct implications for clinical assessment of chelonian blood gas panels, and clearly demonstrates the importance of seasonally-based reference intervals for box turtles, and possibly other ectotherms.

While the pH of blood decreased in the summer months, we did not evaluate the pH of other body compartments in this study. Many aquatic chelonians have elevated levels of bicarbonate in peritoneal/pericardial fluids and high buffering capacity in their shells which allow some species to survive incredible levels of lactic acidosis during prolonged submergence (Jackson, 2000). The degree and speed at which these extravascular buffering sources contribute to changes in arterial and venous pH appears to be species-dependent (Bagatto and Henry, 1999). It is unclear how the buffering capacity of these additional systems may play a role in venous acid–base balance in box turtles, but this could be an avenue for future study.

Blood gas changes associated with PCV mirrored those of temperature, i.e. turtles with higher PCV values had an assumed relative titrational metabolic acidemia. The biological mechanism explaining this finding might be related to hydration status, because elevations in PCV are commonly associated with dehydration during summer months in chelonians (Christopher *et al.*, 1999). Dehydration with a mild decrease in circulatory volume may promote a shift towards anaerobic respiration in the peripheral tissues, driving lactate up and pH down.

Quiet turtles had lower pH, higher pCO_2_ and lower pO_2_ values than bright turtles. This pattern of changes is indicative of a relative respiratory acidemia potentially associated with impaired ventilation in boxed turtles. There were no statistically significant changes in the metabolic component of the blood gas panels, suggesting that the effect of activity level on blood gas parameters is fairly transient compared to some of the other predictor variables evaluated. Taken together, the findings of this study indicate that the clinical interpretation of venous blood gas panels in eastern box turtles should account for changes associated with season, activity level and PCV.

The models constructed for each blood gas parameter contained all of the predictor variables proposed in the DAG, though the inclusion of the activity level variable resulted in a high degree of model selection uncertainty for pO_2_, HCO_3_^−^, TCO_2_ and BE. The models explained a fair-moderate degree of variability in the data, indicating that some important predictor variables may be missing. Heart rate, respiratory rate and plasma electrolyte levels (to calculate anion gap) should be explored as non-invasive measures which may improve the predictive capability of these models. Improvement of proxy variables (such as substituting a more comprehensive metric for activity level) and obtaining dietary history should also be considered in future blood gas modeling efforts.

### Blood gas reference intervals

Reference intervals were constructed for temperature-corrected venous blood gas values. Temperature correction is important for blood gas analyses in ectothermic animals because many portable blood gas analyzers (including the iSTAT) heat blood samples to 37°C prior to analysis, resulting in closed-system changes to the pH, pO_2_ and pCO_2_, as described above. HCO_3_^−^, TCO_2_ and BE are calculated in part using pH and pCO_2_, so these values are also affected by machine methodology. A variety of temperature correction formulae are used to calculate ‘true’ blood gas values from the output of these analyzers for ectotherms (e.g. Stabenau and Heming, 1993; Anderson *et al.*, 2011; Harter *et al.*, 2014; Malte *et al.*, 2014). Some of these formulae are derived from human medicine, while others rely on experimental determination of factors such as αCO_2_, pKa_CO_2__, sodium and protein concentrations, and others to generate species-specific correction formulae. Temperature correction has not been specifically researched for box turtles, and species-specific formulae are not available.

Previous research has demonstrated that human-derived temperature correction formulae provide adequate estimates for pH and pCO_2_ in slider turtles (*Trachemys script*a sp.) and several species of snakes, including ball pythons (*Python regius*), water pythons (*Liasis fuscus*), yellow anacondas (*Eunectes notaeus*) and boas (*Boa constrictor*) (Malte *et al.* 2014). The same study illustrated that human-derived temperature correction formulae for pO_2_ produced substantially biased estimates for reptiles. Based on these results, the present study utilized the iSTAT’s built-in temperature correction formulae for pH and pCO_2_, but relied upon a pO_2_ correction formula from the sea turtle literature (Anderson *et al.* 2011). Following temperature correction of pH, pO_2_ and pCO_2_, values for αCO_2_ and pKa_CO2_ were determined using equations derived for Kemp’s ridley sea turtles (*Lepidochelys kempi*), and HCO_3_^−^ was calculated using an adaptation of the Henderson–Hasselbalch equation (Stabbenau and Heming, 1993). TCO_2_ was calculated using temperature-corrected pCO_2_ and calculated αCO_2_ values. BE is calculated using bicarbonate, pH, and hemoglobin levels. As hemoglobin was not directly measured in this study, BE could not be recalculated using temperature corrected values, and raw iSTAT output is reported.

The use of different temperature correction formulae by different researchers can make direct comparisons between chelonian blood gas studies difficult, however, general observations can still be made. Reference intervals for eastern box turtle blood venous gas parameters were fairly broad and generally consistent with those of apparently healthy, sedated and/or pre-release green sea turtles (*Chelonia mydas*) (Anderson *et al.*, 2011; Lewbart *et al.*, 2014), loggerheads (Chittick *et al.*, 2002; Harms *et al.*, 2003; Camacho *et al.* 2013; Phillips *et al.*, 2017), Kemp’s ridley sea turtles (Innis *et al.*, 2007; Keller *et al.*, 2012), leatherback sea turtles (*Dermochelys coriacea*) (Innis *et al.*, 2010, 2014), hawksbill sea turtles (*Eretmochelys imbricata*) (Muñoz-Pérez *et al.*, 2017), desert tortoises (Christopher *et al.*, 1999; Sleeman *et al.*, 2000; Dennis and Heard, 2002), Negev Desert tortoises (*Testudo werneri*) (Eshar *et al.*, 2014) and diamondback terrapins (*Malaclemys terrapin*) (Christiansen *et al.*, 2013). Reference intervals were partitioned based on season, and parameter estimates are provided in Table S2 to assist with clinical assessment of blood gas panels based on the contributions of other statistically significant predictor variables.

### Limitations and future directions

Limitations of this study are largely related to methodology. The iSTAT is not validated for use in box turtles, and validation of this equipment was beyond the scope of this study. However, previous studies in other ectotherms have identified significant discrepancies in the blood gas values reported by the iSTAT compared to benchtop methods (Harter *et al.*, 2014, 2015; Stoot *et al.*, 2014). One study involving several reptile species demonstrated that iSTAT results for biochemistry parameters were correlated to the results from other analyzers, confirming the clinical utility of this machine with iSTAT-specific reference intervals (McCain *et al.*, 2010). It is considered likely that the results reported from the present study are not truly representative of gold-standard blood gas values, but that they could be related to these values using mathematical transformations once the iSTAT is validated for box turtles (Harter *et al.*, 2014, 2015; Malte *et al.*, 2014).

Lymph dilution is possible from any sampling site in chelonians, but the risk is considered to be higher from the subcarapacial sinus which was used in this study (Hernandez-Divers *et al.*, 2002; Heatley and Russell, 2010). Blood gases were not measured on visually lymph-contaminated samples, however, the possibility of inapparent lymph dilution cannot be ruled out. The timing of blood gas sampling relative to capture can also affect blood gas parameters (Harms *et al.*, 2003, 2016; Innis *et al.*, 2010, 2014). Time of venipuncture relative to time of capture was not recorded in this study, but could be considered as another covariate in future studies involving free-living wildlife.

Directions for future research should include validation of the iSTAT for box turtles and determination of coefficients for temperature correction formulae. The effects of heart rate and respiratory rate on box turtle blood gas parameters should be evaluated, and anion gap should be calculated to better characterize changes in acid–base status.

## Conclusions

This study is a good first step towards understanding blood gas analysis in free-living box turtles. The models established in this study help predict changes in blood gas parameters associated with different physiologic and environmental states, enhancing our ability to distinguish between normal and pathologic variation. The reference intervals can be used for comparison within and between sites in future studies. The ability to comprehensively establish the general wellness of box turtles as sentinel species may also give the clinician a broader understanding of the health of the surrounding ecosystem (Lloyd *et al.*, 2016). Finally, the clinical application of blood gas analysis in the study of disease threats to box turtles may improve conservation strategies for these declining animals.

## Supplementary Material

Supplementary Data
