# Supplementary material for: Venous blood gas in free-living eastern box turtles (Terrapene carolina carolina) and effects of physiologic, demographic and environmental factors
Source: Conserv Physiol. 2018 Jul 25;6(1):coy041. doi: 10.1093/conphys/coy041 (PMC6059089; doi:10.1093/conphys/coy041)

Table S1. Eastern box turtle (*Terrapene carolina carolina*) venous blood gas values excluded from data analysis due to the presence of multiple outlier values from the same individual.

| **ID** | **Season** | **pH** | **pO_2_ (mm Hg)** | **pCO_2_ (mm Hg)** | **HCO_3_^-^ (mmol/L)** | **TCO_2_ (mmol/L)** | **BE (mmol/L)** | **Lactate (mmol/L)** |
| --- | --- | --- | --- | --- | --- | --- | --- | --- |
| 14-1130 | Spring | 7.65 | 73 | 40 | 48.5^a^ | 50.1^a^ | 22^a^ | 2.59 |
| 14-1144 | Spring | 7.79 | 30 | 28 | 62.5^a^ | 64.2^a^ | 23^a^ | 3.58 |
| 14-1604 | Fall | 7.71 | 53 | 41 | 76.9^a^ | 79.3^a^ | 31^a^ | 3.44 |

^a^ Outlier values

Table S2. Parameter estimates with 95% confidence intervals for the effects of environmental and physiologic predictor variables on blood gas parameters in eastern box turtles (*Terrapene carolina carolina*) while controlling for the effects of confounding variables. NS = no statistically significant relationship.

|  | **pH** | **pO_2_**  **(mm Hg)** | **pCO_2_**  **(mm Hg)** | **HCO_3_^-^ (mmol/L)** | **TCO_2_ (mmol/L)** | **Lactate (mmol/L)** | **BE**  **(mmol/L)** |
| --- | --- | --- | --- | --- | --- | --- | --- |
| **Tavg (^o^C)** | **-0.027** | NS | **2.36** | **-0.496** | **-0.441** | **0.058** | **-0.128** |
|  | (-0.036, -0.018) |  | (1.69, 3.02) | (-0.747, -0.245) | (-0.687, -0.194) | (0.032, 0.084) | (-0.209, -0.047) |
| **PCV (%)** | **-0.014** | **-0.951** | **0.968** | **-0.343** | **-0.308** | **0.17** | **-0.342** |
|  | (-0.02, -0.009) | (-1.52, -0.386) | (0.579, 1.36) | (-0.475, -0.212) | (-0.437, -0.178) | (0.087, 0.252) | (-0.48, -0.204) |
| **Activity (Quiet)** | **-0.194** | **-9.26** | **15** | NS | NS | NS | NS |
|  | (-0.115, -0.273) | (-16.97, -1.55) | (9.28, 20.73) |  |  |  |  |
| **Spring* vs. Summer** | **-0.128** | NS | **10.01** | NS | NS | **2.36** | **-3.07** |
|  | (-0.211, -0.045) |  | (3.86, 16.16) |  |  | (3.67, 1.05) | (-0.89, -5.26) |
| **Summer* vs. Fall** | **0.224** | NS | **-17.49** | **4.37** | **3.89** | **-2.72** | **4.71** |
|  | (0.117, 0.330) |  | (9.66, 25.33) | (1.68, 7.06) | (1.29, 6.48) | (-1.01, -4.43) | (7.47, 1.96) |
| **Fall* vs. Spring** | NS | NS | NS | NS | NS | NS | NS |
|  |  |  |  |  |  |  |  |

Figure S1. Histograms of venous blood gas values used to determine reference intervals in eastern box turtles (*Terrapene carolina carolina*) during the spring.


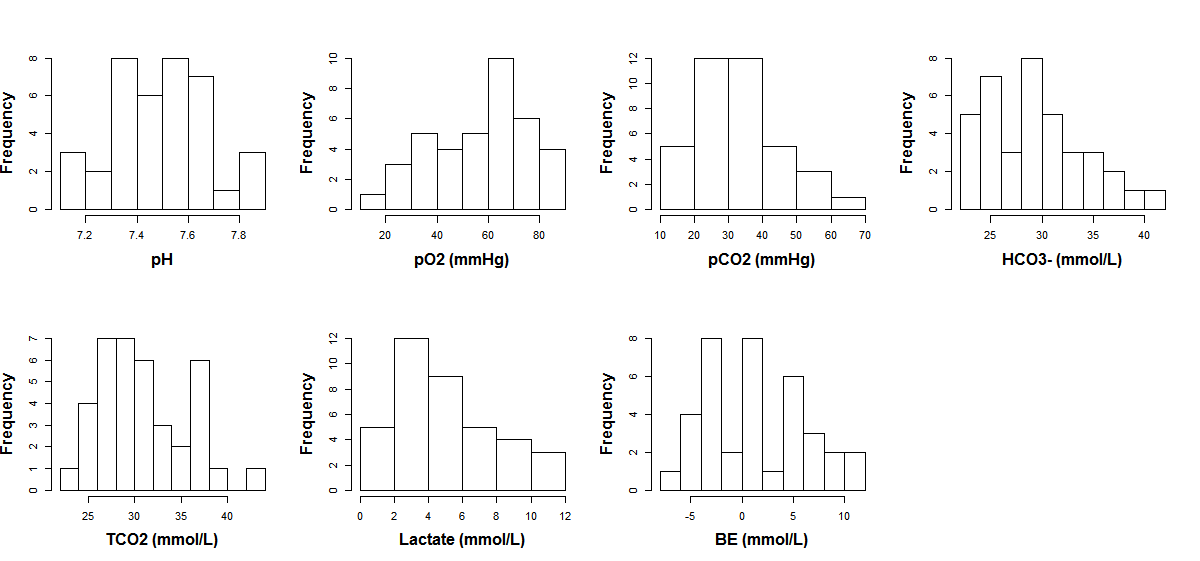


Figure S2. Histograms of venous blood gas values used to determine reference intervals in eastern box turtles (*Terrapene carolina carolina*) during the summer.


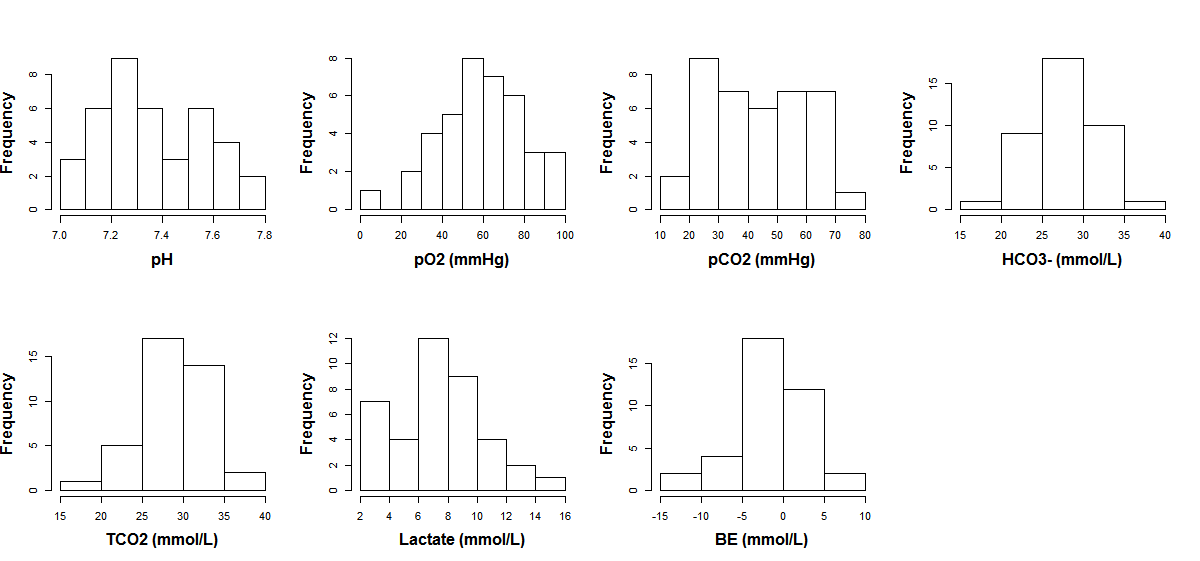


Figure S3. Histograms of venous blood gas values from eastern box turtles (*Terrapene carolina carolina*) during the fall.


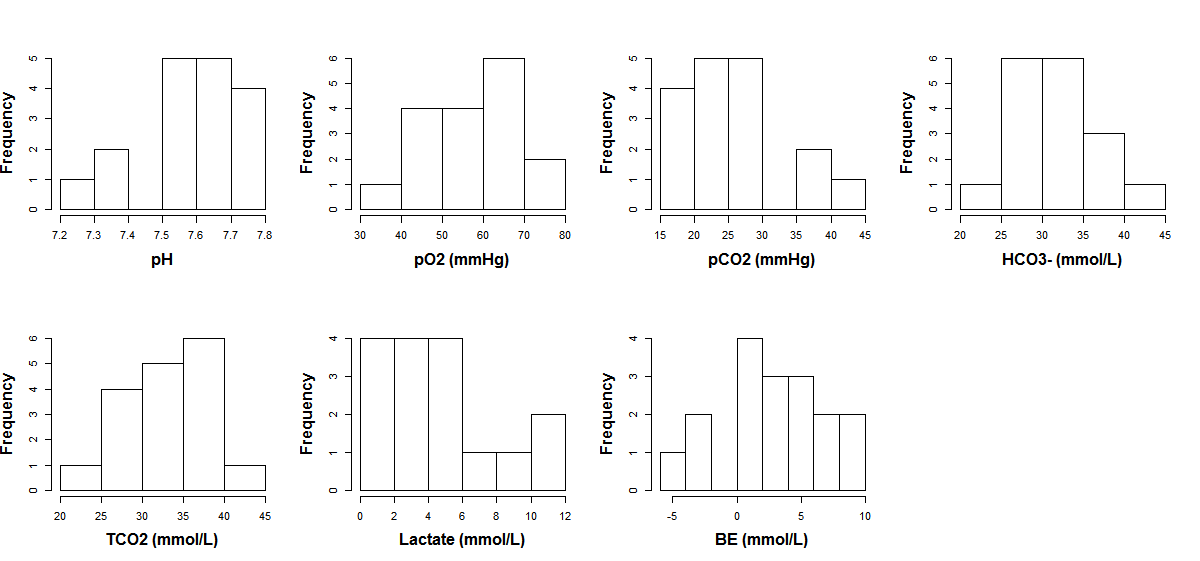

Supplement: Supplementary Data [file coy041_ebt_obt_blood_gas_manuscript_suppl.docx]
